# Investigating the impact of visual perspective in a motor imagery-based brain-robot interaction: A pilot study with healthy participants

**DOI:** 10.3389/fnrgo.2023.1080794

**Published:** 2023-03-30

**Authors:** Andrea Farabbi, Patricia Figueiredo, Fabiola Ghiringhelli, Luca Mainardi, Joao Miguel Sanches, Plinio Moreno, Jose Santos-Victor, Athanasios Vourvopoulos

**Affiliations:** ^1^B3Lab, Dipartimento di Elettronica, Informazione e Bioingegneria (DEIB), Politecnico di Milano, Milan, Italy; ^2^Institute for Systems and Robotics-Lisboa, Instituto Superior Tecnico, Lisbon, Portugal

**Keywords:** Brain-ComputerInterfaces, electroencephalography, motor-imagery, human-robot interaction, neurorehabilitation

## Abstract

**Introduction:**

Motor Imagery (MI)-based Brain Computer Interfaces (BCI) have raised gained attention for their use in rehabilitation therapies since they allow controlling an external device by using brain activity, in this way promoting brain plasticity mechanisms that could lead to motor recovery. Specifically, rehabilitation robotics can provide precision and consistency for movement exercises, while embodied robotics could provide sensory feedback that can help patients improve their motor skills and coordination. However, it is still not clear whether different types of visual feedback may affect the elicited brain response and hence the effectiveness of MI-BCI for rehabilitation.

**Methods:**

In this paper, we compare two visual feedback strategies based on controlling the movement of robotic arms through a MI-BCI system: 1) first-person perspective, with visual information that the user receives when they view the robot arms from their own perspective; and 2) third-person perspective, whereby the subjects observe the robot from an external perspective. We studied 10 healthy subjects over three consecutive sessions. The electroencephalographic (EEG) signals were recorded and evaluated in terms of the power of the sensorimotor rhythms, as well as their lateralization, and spatial distribution.

**Results:**

Our results show that both feedback perspectives can elicit motor-related brain responses, but without any significant differences between them. Moreover, the evoked responses remained consistent across all sessions, showing no significant differences between the first and the last session.

**Discussion:**

Overall, these results suggest that the type of perspective may not influence the brain responses during a MI- BCI task based on a robotic feedback, although, due to the limited sample size, more evidence is required. Finally, this study resulted into the production of 180 labeled MI EEG datasets, publicly available for research purposes.

## 1. Introduction

Worldwide, there is an increasing need for new rehabilitation approaches worldwide for people who suffer from neurological disorders, resulting into chronic motor disability. Every year, between 250,000 and 500,000 people are affected by spinal cord injuries (WHO, 2013), while others are affected by diseases that interrupt the normal communication between the central and the peripheral nervous system, such as brain injuries and cardiovascular disorders, like ischemic and hemorrhagic strokes or transient ischemic attacks (TIA). According to the Global Burden of Disease collaborators for strokes, there are over 13.7 million new strokes each year (GBD, 2019). Neurological rehabilitation is targeting on aims to maximize the restoration of the lost functions of impaired people by inducing neuroplastic changes to the brain (Cauraugh and Summers, 2005), through intensive and repetitive training.

Among the different techniques adopted to improve the effectiveness of this type of rehabilitation, an important effect is starting to be seen by the utilization of Brain-Computer Interfaces (BCI's) (Bamdad et al., 2015). BCI's allow severely impaired patients to interact with the external environment through a computer system by using their brain activity alone, usually measured through electroencephalography (EEG) (Wolpaw and Wolpaw, 2012). The main objective is to promote the recruitment of selected brain areas involved and to facilitate neural plasticity, exploiting the BCI ability of recording and decoding the signals yielded by patient cerebral activity. Specifically, these signals can be reinforced by BCI feedback, so they can be used to strengthen key motor pathways that are thought to support motor recovery after stroke (Birbaumer, 2009; Cervera et al., 2018). Currently, this interaction loop can be closed by controlling an end-effector that includes a simple screen-based feedback (Pfurtscheller et al., 2003), Virtual Reality (VR) (Vourvopoulos et al., 2019), Robotic devices (Tonin and Millán, 2021), or functional electrical stimulation (FES) (Biasiucci et al., 2018).

BCI's can be based on different paradigms depending on the type of brain response that is expected to be involved (e.g., evoked potentials or oscillatory processes). For example, motor imagery BCI (MI-BCI) involves the mental rehearsal of movement and is considered a BCI paradigm that evokes motor-related oscillatory processes between the α (8–12 Hz) and β (13–30 Hz) EEG bands (Pfurtscheller and Neuper, 1997). Next, P300 BCI's use evoked potentials that are generated approximately 300 ms after stimulus onset (hence the name P300), elicited using the oddball paradigm, in which low-probability target items are mixed with high-probability non-target items (Polich and Margala, 1997). Finally, the steady-state visually evoked potentials (SSVEPs) are also evoked potentials caused by visual stimulation (e.g., flashing light), which occur at the primary visual cortex of the brain (Creel, 2019). Both P300 and SSVEP BCI paradigms are used mainly as assistive interfaces (e.g., for patients in the locked-in state), while MI-BCI captures activity over the motor and somatosensory cortices and is used primarily for motor restoration and rehabilitation (Cervera et al., 2018).

Specifically, MI shares many of the neural mechanisms with the actual movement (Lotze et al., 1999; Kimberley et al., 2006), including the recruitment of the prefrontal cortex, which is responsible for the creation and maintenance of a clear representation used during action and imagination (Hanakawa et al., 2003). However, the level of the activation of the Central Nervous System (CNS) activation is on average weaker in imaginary movement than in the concrete action. In general, when a neural structure is activated, there is a desynchronization of single neurons activities that is reflected in a decrease in Power Spectral Density (PSD) of the EEG signal in certain frequency ranges; when there is an idle state, instead, there is a synchronization of single neurons activities, leading to an increase in the PSD (Perry and Bentin, 2009). The two phenomena are recognized as event-related desynchronization (ERD) and synchronization (ERS), respectively, and in general are observable between the 8–30 Hz band, within the α (or μ) and β bands (McFarland et al., 2000).

The degree of activation of the cortex has been observed to be linked to the type of imagery performed. There are two types of strategies: kinesthetic motor imagery and visual motor imagery (Guillot et al., 2004). In the first one, the subjects feel that they are actually performing the movement, with coordination and all the related sensory perception, so from a first-person perspective comprehensive of the whole elicited sensations (or the projection of them). Whereas, in the latter, the subjects just observe themselves from an internal (first-person view) or external perspective (third-person view). Stinear et al. observed an effective difference between the two modalities: kinesthetic MI was found to modulate corticomotor excitability. These findings had an impact in terms of rehabilitation purposes (Stinear et al., 2005).

One limitation observed along among various BCI implementations is that not all subjects are able to use an MI-based BCI; this is a commonly reported limitation, referred to as BCI illiteracy (Vidaurre and Blankertz, 2010). Since a BCI-implementation- related experiment requires a relatively long large amount of time and effort, prior studies have attempted to identify factors that could improve performance in MI-BCI control. Some examples include the use of tactile feedback (Pillette et al., 2021), task gamification (Vourvopoulos et al., 2016), motor-priming prior to the MI-BCI training session (Vourvopoulos and Bermúdez i Badia, 2016), but also, and the use of embodied feedback either in the virtual or the physical world through robots. Specifically, prior research has shown that MI skills can be augmented by using humanlike hands (Alimardani et al., 2016), and that the sense of embodiment in VR can be enhanced by multisensory feedback related to body movements (Leonardis et al., 2014).

Despite the large number of studies in this area and the variety of methods adopted to increase the effectiveness of these systems (Lotte et al., 2007, 2018), some methodological issues still remain unclear. For example, in literature, it's it is not clear which kind of feedback, especially in which condition, is most beneficial for the user. Specifically, research on the impact of robot hands' perspective on EEG ERD patterns is relatively limited. A recent survey revealed that a small subset of studies has have investigated BCI in controlling robotic arms (most of the studies involve wheelchairs or drones), and there are some further very limited in studies analyzing ERD patterns (Zhang and Wang, 2021). The majority of the studies report mainly classification accuracies and BCI performance metrics.

The aim of this study is to investigate the impact of the visual perspective (first- vs. third- person perspective) on ERD power, lateralization, and learnability during a Brain-Robot Interaction. To achieve this, a longitudinal study was designed, including wherein 10 healthy participants underwent, undergoing the two MI-BCI conditions in over three consecutive days.

## 2. Methods

### 2.1. Demographics

For this experiment, 12 healthy subjects were recruited; one subject was excluded due to left-handedness for consistency purposes, and one due to a dropout, resulting into a total of 10 participants. The mean age was 25 years (±6), with a balanced sample between 5 males men and 5 females women (Table 1). All No recruited participants had no prior experience with neurofeedback or BCI, and all were without any known neurological disease. All participants signed an their informed consent before participating in the study in accordance with the 1964 Declaration of Helsinki, and the protocol was approved by the Ethics Committee of CHULN and CAML (Faculty of Medicine, University of Lisbon) with reference number: 245/19.

**Table 1:** Subjects demographics.

Number of subjects	10
Gender	5 females, 5 males
Mean age (years)	25 ± 6
Handedness	Right hand dominance
Background	University students (5) and lab staff (5)

### 2.2. Protocol

**Figure 1:**
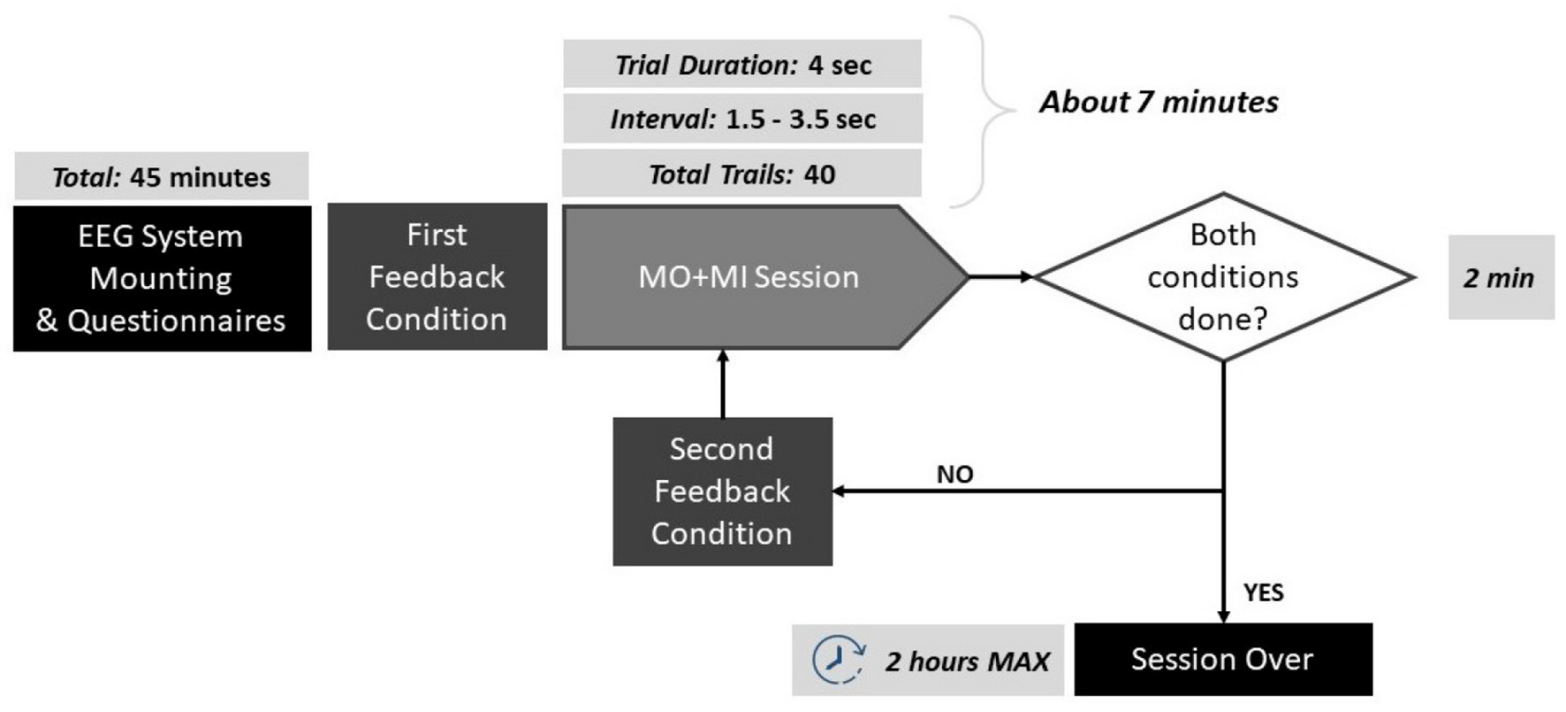
Schematic of the protocol pipeline. The duration of each step is reported: the experiments lasted maximum 2 h per participant.

Volunteers were instructed to stay as still as possible during the MI to avoid any motor- related activity that was not related to the actual task. To facilitate their MI performance, a simple MI task was chosen that involved reaching an object on the table in front of them, moving the right or the left arm. During trials, subjects were asked to imagine the movement of their arm in a kinesthetic way, including the imagination of the coordination of all the muscles, joints involved in the real action, and the relative sensations. As feedback provision, the robot was moving its arms forward to reach the object and then going back, continuously, resembling the imagined movement. The complete protocol pipeline is illustrated in Figure 1.

The MI trials were based on the Graz paradigm (Pfurtscheller et al., 2003), and can be described as follows: each trial lasted 4 s, forewarned by the appearance of a green cross on the screen and a concomitant beep-sound a second before the onset of the task. The beep sound was added in order to prepare the subject for the incoming trial. Next, an arrow was appearingappeared pointing right or left, and the subject had to imagine the continuous movement of the corresponding arm reaching for the object for as long as the green cross was on. To each subject, 20 arrows pointing to the left and 20 pointing to the right were displayed randomly, for a total of 40 trials. Between each trial, there was an interval that lasted randomly between 1.5 and 3.5 s. The robot was moving the corresponding hand, depending on the arrow direction, and for the entire of the duration. Since the robot was moving during the training trials, the elicited brain activity can be described as a mixture of MI and Motor Observation (MO).

### 2.3. Experimental setup

**Figure 2:**
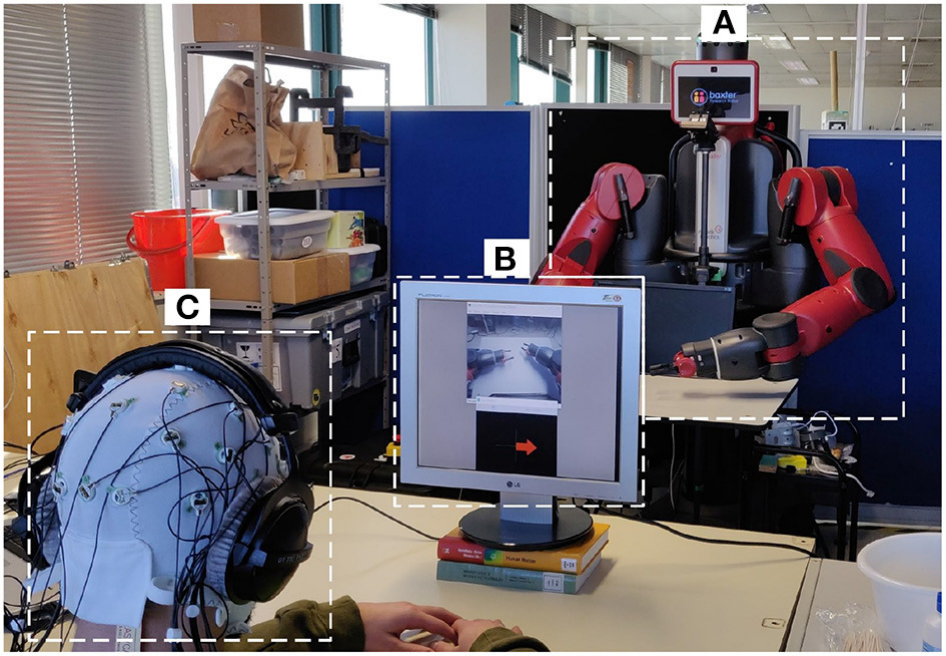
Experimental setup. **(A)** The Baxter robot; **(B)** screen for delivering the visual simulations to the user for the first-person perspective; **(C)** participant with the 32 electrode EEG system and headphones for auditory stimulation and impeding outside noise.

This experiment was performed in a laboratory environment under controlled conditions. The subjects went through two BCI acquisitions (one per condition) lasting a maximum of 2 h, during over three consecutive days, each day at approximately the same hour of the day. This was selected to minimize confounding factorss related to the time of the acquisition of the EEG. During the session, subjects were comfortably sited seated on a chair with their arms resting on a table (Figure 2).

#### 2.3.1. The robot

The robot used was Baxter (Rethink Robotics, Bochum, Germany), a versatile research robot, 2 m tall, 140 kg of in weight, with a wheeled pedestal that allows mobility (Figure 2A). It presents an anthropomorphic design with its animated face and two arms, in which the hands are replaced by two grips to permit allow it to grab tools. Each limb has 7 degrees of freedom (DoF) that make it able to perform continuous and natural movements. Baxter is using an open-source Robot Operating System (ROS) (Quigley et al., 2009), and it provides a stand-alone server (i.e., ROS Master) to which any development workstation can connect and control Baxter ROS *via* the various ROS Application Programming Interface (APIs).

#### 2.3.2. Visual feedback

For the first-person perspective condition, a camera was mounted at the head of the robot, capturing the robot arms and displaying them in a secondary computer monitor, together with the MI instructions (Figure 2B). For the third-person perspective, the secondary monitor was removed, and the MI instructions were delivered directly from a monitor attached in front of the robot, so the robot hand was in front of the field-of-view of the participant (Figure 2A).

#### 2.3.3. Instrumentation

For the EEG acquisition, the LiveAmp 32 (Brain Products, Gilching, Germany) system was used. LiveAmp is a wireless amplifier with 32 active electrodes arranged in the 10-20 standard configuration, and a triaxial accelerometer (Figure 2C). The EEG signals were sampled at 250 Hz.

### 2.4. Software for analysis

For acquisition and visualization purposes, the OpenVibe software platform was used. OpenVibe, enables to the design, testing, and use of BCI pipelines through an intuitive platform consisted consisting of a set of software modules (in C++) that can be easily and efficiently integrated to design BCI (Renard et al., 2010). To analyze the data, MATLAB was used with the EEGLAB toolbox (Delorme and Makeig, 2004), a dedicated plugin for processing and analyzing continuous and event-related EEG. It permits to workallows for working with EEG data at an individual level and with a group study.

### 2.5. Preprocessing

The acquired signal was preprocessed in order to enhance the brain activity of interest and to lower the present noise. First, the EEG signal was band-pass filtered between 1 and 40 Hz in order to remove high- frequency noise usually related to muscle artifacts and not of interest in MI tasks. Then, channels were re-referenced to Common Average Reference (CAR), after the identified bad channels were interpolated in order to not spread the noise of the bad channels to the others. After this, the epochs of interest (MI related to left and right movement imaginations) were extracted and, Independent Component Analysis (ICA) was then employed in order to remove eyes movement, blinking, and muscle- related artifacts.

### 2.6. EEG data analysis

To investigate the differences in terms of conditions and the evolution of the brain response along sessions, a group study was performed on the recorded data. In particular, the EEG signal acquired during trials was analyzed both in the temporal and spatial domain. Concerning the temporal analysis, the Event-Related Desynchronization (ERD) in the α band (*8-12Hz*) was computed for each trial and each subject, since it is related with MI (Babiloni et al., 1999). This feature was extracted from the Event-Related Spectral Perturbation (ERSP), where the PSD of a single epoch is computed both across time and in the frequency range of interest through time-frequency decomposition using Morlet wavelets. The PSD was estimated using the Welch method. The computed ERSP was divided by a baseline, represented by the ERSP computed in the second before the stimulus onset. Concerning the spatial domain, a qualitative and a quantitative analysis were performed. For this purpose, the ERD was averaged across time and represented through scalp topographical plots. This was done in order to qualitatively assess any differences in the spatial distribution of the ERD. Regarding the contralateral/ipsilateral activation during left and right epochs, the ERD lateralization was extracted through the Lateralization Index (LI) (Doyle et al., 2005), and has been computed using the following formula:


(1) $$\begin{array}{l} LI=\frac{\left(ERDC3_{Left}-ERDC4_{Left}\right)+\left(ERDC4_{Right}-ERDC3_{Right}\right)}{2} \end{array}$$

where C3 and C4 represent the electrodes examined and left and right correspond to the arm that the user was supposed to imagine to movinge. The LI value can be interpreted as follows: ipsilateral dominance corresponds to a negative value, while contralateral one to positive LI. Indeed, taking into account just a single task analysis (left or right), if the contralateral value is lower than the ipsilateral value, meaning a contralaterally desynchronized status, LI would be positive. LI has been computed for every session and condition, both for the training and online phases, in α band.

### 2.7. Statistical analysis

Comparison between conditions was performed through statistical tests. Since data distributions were not normal, but also due to the small sample size, non-parametric tests were employed.

In particular, the Friedman test was used as the non-parametric alternative to the Repeated Measures ANOVA for finding any statistically significant differences among conditions, both in terms of ERDs and LIs. Moreover, the Friedman Test was also used to assess any learning effect through a comparison between the first and last sessions for each condition. or all statistical comparisons; the significance level was set to 5% (*p* < 0.05) and were was carried out using MATLAB R2021a.

## 3. Results

In this section, we report the ERD of the α band, and over the C3 and C4 electrodes since they are the target electrodes for acquiring sensorimotor rhythms.

### 3.1. Impact of visual perspective

**Figure 3:**
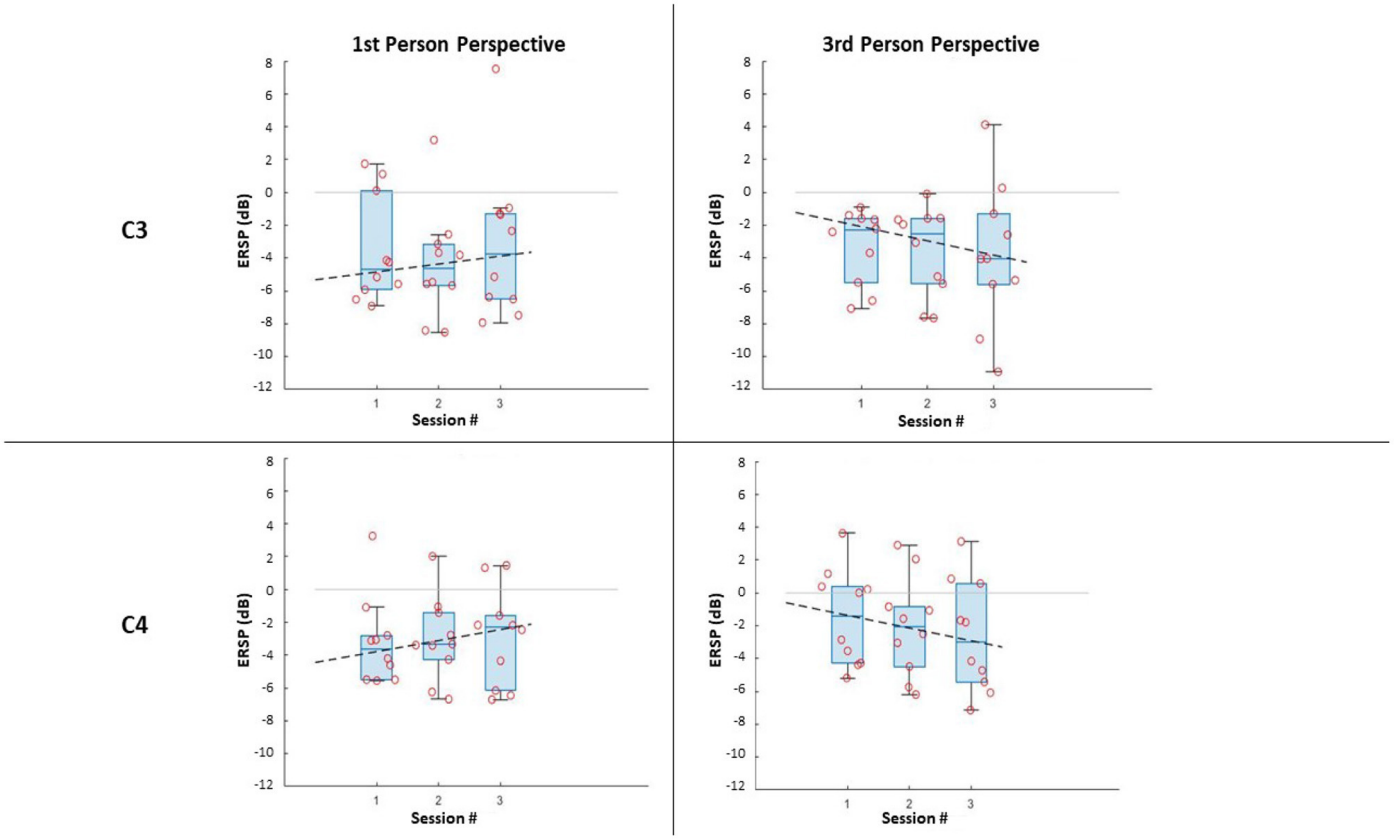
ERSP distributions along sessions for MI right-hand trials: ERSP recorded in channel C3 and C4 in the first-person and third-person perspectives. The dashed lines represent the interpolation of the medians of each session.

**Figure 4:**
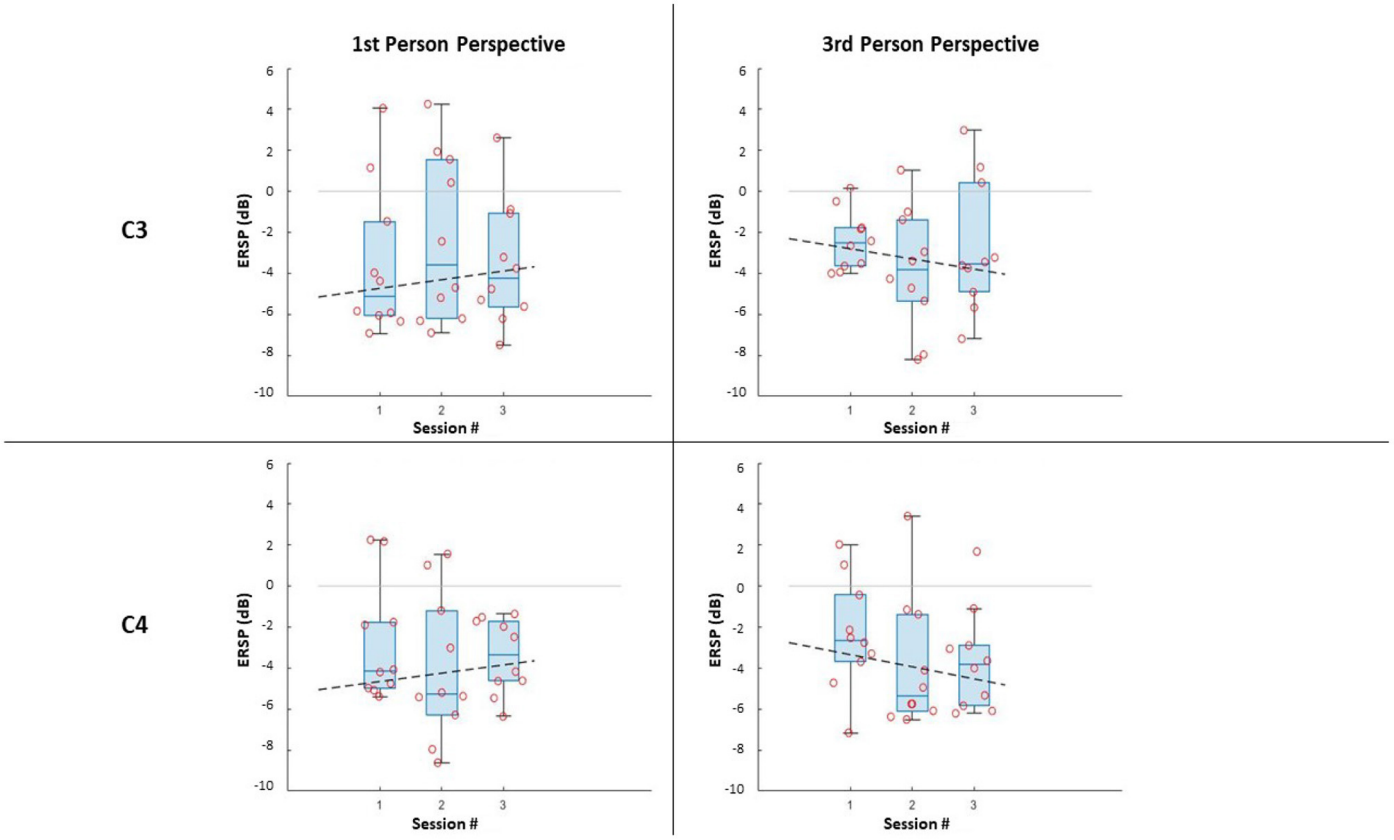
ERSP distributions along sessions for MI left-hand trials: ERSP recorded in channel C3 and C4 in the first-person and third-person perspectives. The dashed lines represent the interpolation of the medians of each session.

In terms of the impact of the visual perspective, the Friedman test yielded no significant differences between conditions, for all sessions, for both electrodes (C3; C4), and for both right (Figure 3) and left (Figure 4) trials. The χ^2^ scores and *p*-values obtained by this comparison are reported in Table 2.

**Table 2:** The χ^2^ score and the *p*-values obtained through the Friedman test comparing the two different perspective conditions.

	**C3**						**C4**					
	**Left**			**Right**			**Left**			**Right**		
	**Ses 1**	**Ses 2**	**Ses 3**	**Ses 1**	**Ses 2**	**Ses 3**	**Ses 1**	**Ses 2**	**Ses 3**	**Ses 1**	**Ses 2**	**Ses 3**
$\chi_{\left(1\right)}^{2}$	0.4	1.6	1.6	0.4	1.6	0.4	1.6	0.4	0.4	1.6	1.6	1.6
*p*-value	0.527	0.206	0.206	0.527	0.206	0.527	0.206	0.527	0.527	0.206	0.206	0.206

None of the obtained values showed significant differences.

Further, it can be noticed that, in all cases, most of the subjects managed to evoke ERD during MI, resulting in negative values of ERSPs (α desynchronization), with only a few outliers. It is worth noticing that the desynchronization in the two electrodes (C3; C4), is not only similar for both conditions, but also for the same side (Left;Right MI).

**Figure 5:**
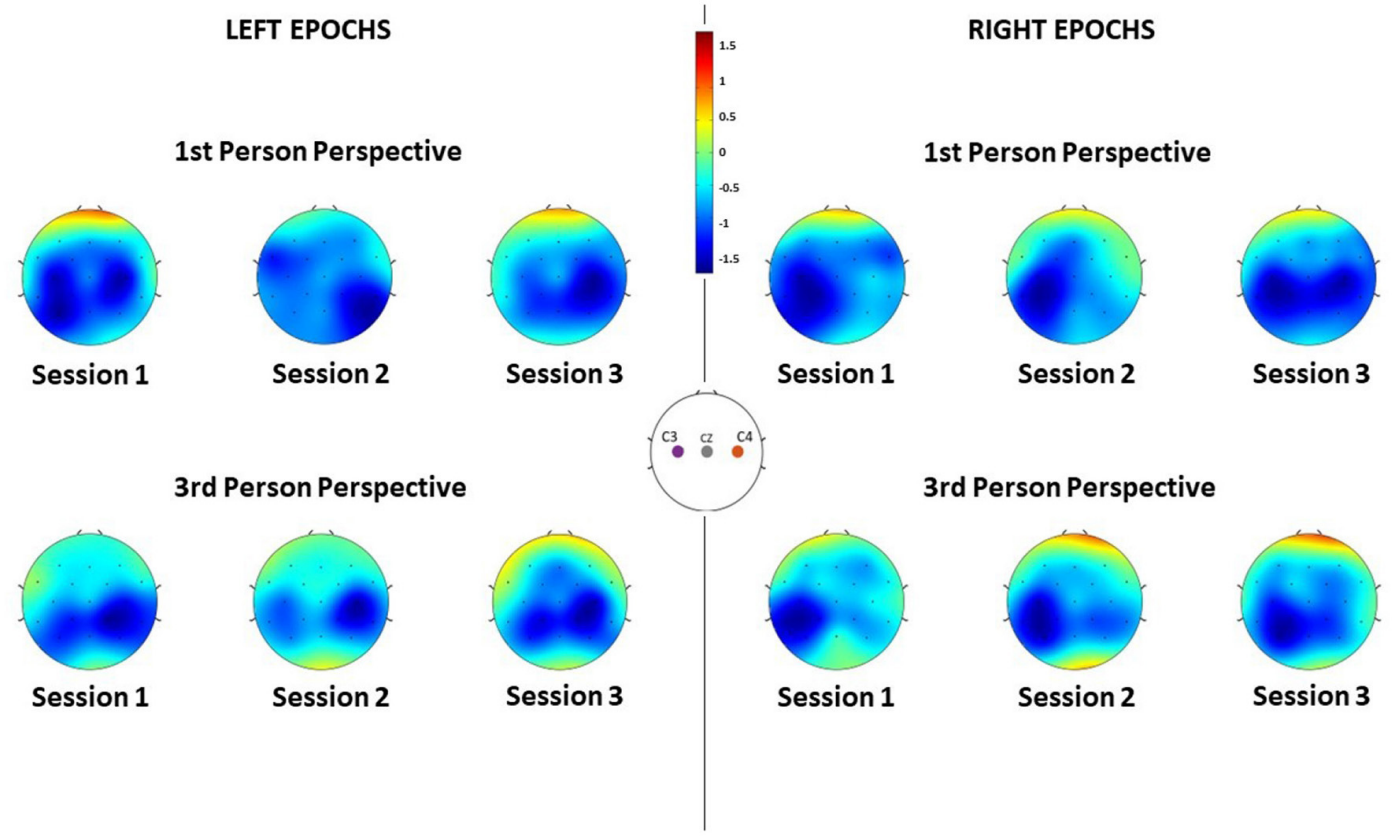
ERD of each channel averaged along subjects during the different sessions, for each feedback condition and for both left and right MI epochs. Negative values indicate desynchronization of that region.

Concerning the spatial distribution of the α desynchronization, the most activated region is the sensorimotor area, around C3 and C4 electrodes as anticipated (Figure 5). However, it is worth noticing that the brain activation is actually spread in throughout both the electrodes C3 and C4, mainly in Session 1 (Left epochs) and Session 3 (Right epochs). In all other cases, a more contralateral activation is observed, mainly from the third-person perspective condition. Nonetheless, the spatial patterns are similar between conditions.

**Figure 6:**
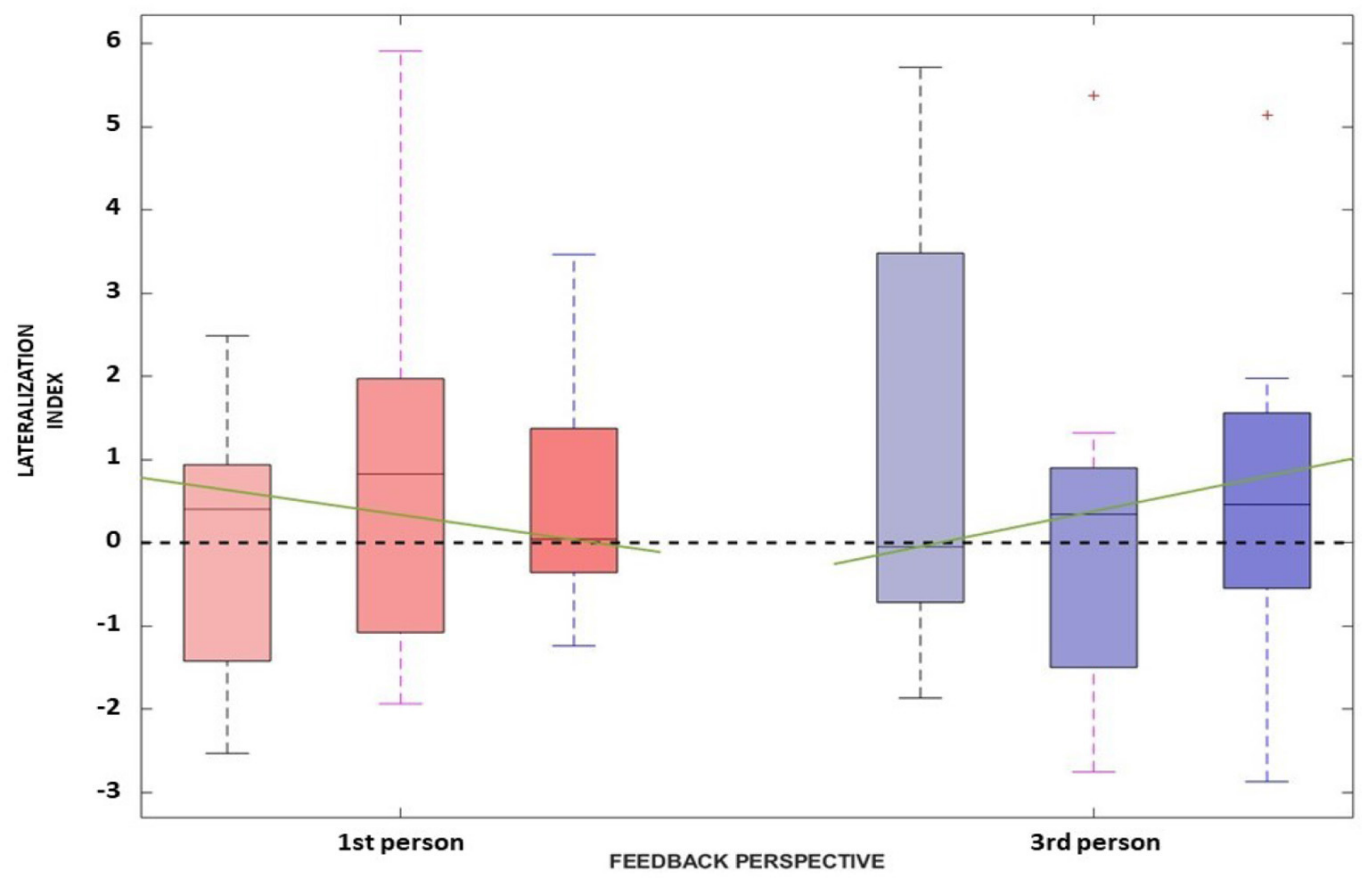
The Lateralization Index distributions along the three sessions for the *first-person perspective* (in red) and *third-person perspective* (in blue) are reported. The green line represents the interpolation of the medians obtained in the three sessions for each feedback condition.

This is also confirmed also by the LI distributions along sessions (Figure 6) where no significant difference has been found between the first- and third- person perspective [i.e., χ^2^(1) and *p-values* for each session are: (1.6, 0.206), (1.6, 0.206), and (1.6, 0.206)]. Overall, it is not possible to verify any predominance of the laterality for both visual conditions.

### 3.2. Impact of time-learning effect

In this section, we are assess if the ERSP is changing across the three different sessions, indicating possible learning effects.

The comparison along sessions concerning the ERSP metric did not yield any significant difference across time in both conditions for both electrodes and epochs. The results of the Friedman test in terms of χ^2^ score and *p-values* are reported in Table 3. However, the interpolation of the medians of the ERSPs distributions of each session (Figures 3, 4) showed two different behaviors for the two feedback conditions: the *first-person perspective* resulted on in an increasing trend of ERSP along the three sessions for both electrodes and epochs, while the *third-person perspective* resulted in a decreasing trend of decrease along sessions. The slopes related to this trend are reported in Table 3.

**Table 3:** Statistics obtained for the comparison between sessions in terms of Lateralization Index.

	**C3**				**C4**			
	**Left**		**Right**		**Left**		**Right**	
	**1st PP**	**3rd PP**	**1st PP**	**3rd PP**	**1st PP**	**3rd PP**	**1st PP**	**3rd PP**
$\chi_{\left(1\right)}^{2}$	1.6	1.6	0.4	0.4	1.6	1.6	0.4	1.6
*p*-value	0.206	0.206	0.527	0.527	0.206	0.206	0.527	0.206
Slope	0.42	–0.50	0.48	–0.87	0.40	–0.59	0.67	–0.77

The χ^2^ score and the p-value of the comparison between first and third session and the slope of the trend of the medians obtained during the three sessions are reported.

Concerning the LI, a similar behavior can be assessed. Again, the Friedman test did not yield any significant differences between the first- [$\chi_{\left(1\right)}^{2}=0.4$, *p* = 0.527] and third- [$\chi_{\left(1\right)}^{2}=1.6$, *p* = 0.206] person perspective across sessions, respectively. Moreover, the trend along sessions, as extracted by the median slopes, show two different behaviors depending on the feedback condition. Specifically, the first-person perspective has a negative trend over time (*coef*:−1.193), while the third-person perspective resulted in a positive trend (*coef*:1.695) as illustrated in Figure 6.

## 4. Discussion

The obtained results suggest that in all visual perspective conditions during Brain-Robot Interaction through MI, we can elicit α desynchronization responses of the somatosensory area. Nonetheless, no impact of the condition nor time was found on the α ERSP power. Moreover, the α ERSP distribution at scalp level showed no predominant hemisphere, with the spread all over the somatosensory area, highlighting no ipsilateral or contralateral dominance. However, during the *third-person perspective* condition of feedback, the ERD seems to be more contralaterally localized than the one observed during the *first-person perspective*, where, especially in the third session, electrodes C3 and C4 recorded a similar activity. The contralateral α ERSP during the *third-person perspective* feedback can be particularly noticed during the MI of the right hand, which and that can be related to the fact that all the participants analyzed had right-hand dominance, thus it would be easier for them to imagine vividly the movement of their dominant hand.

Concerning ERSP development over time, we noticed that the two feedback conditions gave two different behaviors along sessions, when interpolating the medians of the ERSP distributions. This trend was used to assess the learning ability of the subjects during the ongoing of throughout the experiment. In particular with learning ability, we intend the capability aim to have a more vivid imagination of the task investigated, resulting in a more desynchronized response of the neurons (i.e., the ERSP decreases across time). The *first-person perspective* condition trend along the three session increases, while the one for the *third-person perspective* decreases, suggesting a better learning ability of the subject with this type of feedback. However, a consideration must be done made for the *first-person perspective* condition. The use of the monitor for simulating this condition is not optimal, since it does not give the right sense of embodiment to the subjects. It is suggested to use different means for replicative of this perspective in different ways, such as by using visors for Virtual Reality, that which are is already widely used in MI-based BCI trials (Vourvopoulos et al., 2019).

Moreover, both for the spatial and temporal domain analyses, no statistically significant differences were highlighted between sessions. This may be due to the restricted number of participants and, we believe, to the number of sessions (and the interval between them) that does not permit to strongly assess the presence of learning ability of the subjects along across sessions.

Concerning the direct comparison between the two perspective conditions, the Friedman test on the metrics of interest revealed no significant differences. This suggests that the two conditions do not affect the brain response during MI tasks, but, again, it must be taken into account that the simulation of the *first-person perspective* was not optimal in the proposed protocol.

Overall, current findings contribute for to the future development of robot-assisted rehabilitation, given the impact that MI BCI's could have in re-training the lost functions in stroke patients, and promotinge recovery by employing intensive and repetitive motor training (AL-Quraishi et al., 2018). Furthermore, the latest research in rehabilitation robotics highlighted the importance that embodied robotic feedback could offer in lasting clinical outcomes (Robinson et al., 2021), while robotic-assisted upper limb systems allow the performance of tasks of everyday living (e.g., grasping, eating, and personal hygiene), found to be amongst the most important activities to be exercised in rehabilitation (Rätz et al., 2022).

## 5. Conclusions

In this paper, a study of the impact of the visual perspective of robotic arms on brain response during MI tasks is presented. The results obtained in terms of temporal and spatial activation did not indicate any significant differences, suggesting that the robot arm perspective given to the subject during MI trials does not affect the elicited activity of the brain in terms of α ERSP. However, this is a preliminary study, since a general effect on the learning ability of the subjects was observed in the analyzed ERSP, but, due to the restricted number of sessions and subjects, the considerations about it are limited. Future development of this work should consider increasing the number of sessions and the number of participants and, moreover, to changinge the method of presenting the *first-person perspective*, and improvinge the perceived sense of embodiment of the robotic limbs. Last but not least, this study also resulted into the production of 180 labeled MI EEG datasets during robot arm movement, publicly available for educational and research purposes (Farabbi et al., 2020).

## Data availability statement

The datasets presented in this study can be found in online repositories. The names of the repository/repositories and accession number(s) can be found below: Zenodo repository: https://zenodo.org/record/5882500.

## Ethics statement

The studies involving human participants were reviewed and approved by Ethics Committee of CHULN and CAML (Faculty of Medicine, University of Lisbon) with reference number: 245/19. The patients/participants provided their written informed consent to participate in this study.

## Author contributions

AF, PF, FG, LM, JS, and AV defined and designed the research study. AF and FG participated in the development of the software and performed the participant recruitment and assessments, and collected the data. PM and JS-V provided and assisted with the Robot hardware. AF analyzed the data and compiled the manuscript. AF, PF, and AV interpreted the data. All authors revised and approved the final version of the manuscript.

## References

[B1] Alimardani M. Nishio S. Ishiguro H. (2016). The importance of visual feedback design in bcis; from embodiment to motor imagery learning. PLoS ONE 11, e0161945. 10.1371/journal.pone.0161945 27598310 PMC5012560

[B2] AL-Quraishi M. S. Elamvazuthi I. Daud S. A. Parasuraman S. Borboni A. (2018). Eeg-based control for upper and lower limb exoskeletons and prostheses: a systematic review. Sensors 18, 3342. 10.3390/s18103342 30301238 PMC6211123

[B3] Babiloni C. Carducci F. Cincotti F. Rossini P. M. Neuper C. Pfurtscheller G.. (1999). Human movement-related potentials vs desynchronization of eeg alpha rhythm: a high-resolution eeg study. Neuroimage 10, 658–665. 10.1006/nimg.1999.0504 10600411

[B4] Bamdad M. Zarshenas H. Auais M. A. (2015). Application of bci systems in neurorehabilitation: a scoping review. *Disabil. Rehabil*. Assist. Technol. 10, 355–364. 10.3109/17483107.2014.961569 25560222

[B5] Biasiucci A. Leeb R. Iturrate I. Perdikis S. Al-Khodairy A. Corbet T.. (2018). Brain-actuated functional electrical stimulation elicits lasting arm motor recovery after stroke. Nat. Commun. 9, 1–13. 10.1038/s41467-018-04673-z 29925890 PMC6010454

[B6] Birbaumer N. E. A. (2009). Neurofeedback and brain-computer interface clinical applications. Int. Rev. Neurobiol. 86, 107–117. 10.1016/S0074-7742(09)86008-X 19607994

[B7] Cauraugh J. H. Summers J. J. (2005). Neural plasticity and bilateral movements: a rehabilitation approach for chronic stroke. Progr. Neurobiol. 75, 309–320. 10.1016/j.pneurobio.2005.04.001 15885874

[B8] Cervera M. A. Soekadar S. R. Ushiba J. Millán J. d R Liu M.. (2018). Brain-computer interfaces for post-stroke motor rehabilitation: a meta-analysis. Ann. Clin. Transl. Neurol. 5, 651–663. 10.1002/acn3.544 29761128 PMC5945970

[B9] Creel D. J. (2019). Visually evoked potentials. Handb Clin. Neurol. 160, 501–522. 10.1016/B978-0-444-64032-1.00034-5 31277872

[B10] Delorme A. Makeig S. (2004). Eeglab: an open source toolbox for analysis of single-trial eeg dynamics including independent component analysis. J. Neurosci. Methods 134, 9–21. 10.1016/j.jneumeth.2003.10.009 15102499

[B11] Doyle L. M. Yarrow K. Brown P. (2005). Lateralization of event-related beta desynchronization in the EEG during pre-cued reaction time tasks. Clin. Neurophysiol. 116, 1879–1888. 10.1016/j.clinph.2005.03.017 15979401

[B12] Farabbi A. Ghiringhelli F. Mainardi L. Sanches J. M. Moreno P. Santos-Victor J.. (2020). Motor-Imagery EEG Dataset During Robot-Arm Control. Available online at: https://zenodo.org/record/5882500

[B13] GBD (2019). Global, regional, and national burden stroke, 1990–2016: a systematic analysis for the global burden of disease study 2016. Lancet Neurol. 18, 439–458. 10.1016/S1474-4422(19)30034-1 30871944 PMC6494974

[B14] Guillot A. Collet C. Dittmar A. (2004). Relationship between visual and kinesthetic imagery, field dependence-independence, and complex motor skills. J. Psychophysiol. 18, 190–198. 10.1027/0269-8803.18.4.190

[B15] Hanakawa T. Immisch I. Toma K. Dimyan M. A. Gelderen P. V. Hallett M. (2003). Functional properties of brain areas associated with motor execution and imagery. J. Neurophysiol. 89, 989–1002. 10.1152/jn.00132.2002 12574475

[B16] Kimberley T. Khandekar G. Skraba L. Spencer J. Gorp E. V. Walker S. (2006). Neural substrates for motor imagery in severe hemiparesis. Neurorehabil Neural Repair. 20, 268–277. 10.1177/1545968306286958 16679504

[B17] Leonardis D. Frisoli A. Barsotti M. Carrozzino M. Bergamasco M. (2014). Multisensory feedback can enhance embodiment within an enriched virtual walking scenario. Presence 23, 253–266. 10.1162/PRES_a_00190

[B18] Lotte F. Bougrain L. Chichokia A. Clerk M. Congedo M. Rakotomamonjy A.. (2007). A review of classification algorithms for eegbased brain-computer interfaces. J. Neural Eng. 4, R1–R13. 10.1088/1741-2560/4/2/R01 17409472

[B19] Lotte F. Bougrain L. Chichokia A. Clerk M. Congedo M. Rakotomamonjy A.. (2018). A review of classification algorithms for eeg-based brain-computer interfaces: a 10 year update. J. Neural Eng. 15, aab2f2. 10.1088/1741-2552/aab2f2 29488902

[B20] Lotze M. Montoya P. Erb M. Hülsmann E. Flor H. Klose U.. (1999). Activation of cortical and cerebellar motor areas during executed and imagined hand movements: an fmri study. J. Cogn. Neurosci. 11, 491–501. 10.1162/089892999563553 10511638

[B21] McFarland D. Miner L. Vaughan T. M. Wolpaw J. R. (2000). Mu and beta rhythm topographies during motor imagery and actual movements. Brain Topogr. 12, 177–186. 10.1023/A:1023437823106 10791681

[B22] Perry A. Bentin S. (2009). Mirror activity in the human brain while observing hand movements: a comparison between eeg desynchronization in the μ-range and previous fmri results. Brain Res. 1282, 126–132. 10.1016/j.brainres.2009.05.059 19500557

[B23] Pfurtscheller G. Neuper C. (1997). Motor imagery activates primary sensorimotor area in humans. Neurosci. Lett. 239, 65–68. 10.1016/S0304-3940(97)00889-6 9469657

[B24] Pfurtscheller G. Neuper C. Muller G. Obermaier B. Krausz G. Schlogl A.. (2003). Graz-bci: state of the art and clinical applications. IEEE Trans. Neural Syst. Rehabil. Eng. 11, 1–4. 10.1109/TNSRE.2003.814454 12899267

[B25] Pillette L. N'kaoua B. Sabau R. Glize B. Lotte F. (2021). Multi-session influence of two modalities of feedback and their order of presentation on mi-bci user training. Multimodal Technol. Interact. 5, 12. 10.3390/mti5030012

[B26] Polich J. Margala C. (1997). P300 and probability: comparison of oddball and single-stimulus paradigms. Int. J. Psychophysiol. 25, 169–176. 10.1016/S0167-8760(96)00742-8 9101341

[B27] Quigley M. Conley K. Gerkey B. Faust J. Foote T. Leibs J.. (2009). Ros: an open-source robot operating system, in ICRA Workshop on Open Source Software, Vol. 3 (Kobe), 5.

[B28] Rätz R. Müri R. M Marchal-Crespo L. (2022). Assessment of clinical requirements for a novel robotic device for upper-limb sensorimotor rehabilitation after stroke, in Converging Clinical and Engineering Research on Neurorehabilitation IV: Proceedings of the 5th International Conference on Neurorehabilitation (ICNR2020), October 13-16, 2020 (Cham: Springer), 171–175. Available online at: https://link.springer.com/chapter/10.1007/978-3-030-70316-5_28

[B29] Renard Y. Lotte F. Gibert G. Congedo M. Maby E. Delannoy V.. (2010). Openvibe: an open-source software platform to design, test, and use brain-computer interfaces in real and virtual environments. Presence 19, 35–53. 10.1162/pres.19.1.35

[B30] Robinson N. Mane R. Chouhan T. Guan C. (2021). Emerging trends in bci-robotics for motor control and rehabilitation. Curr. Opin. Biomed. Eng. 20, 100354. 10.1016/j.cobme.2021.10035414624080

[B31] Stinear C. M. Byblow W. D. Steyvers M. andStephan P. Swinnen O. L. (2005). Kinesthetic, but not visual, motor imagery modulates corticomotor excitability. Exp. Brain Res. arch. 168, 157–164. 10.1007/s00221-005-0078-y 16078024

[B32] Tonin L. Millán J. d. R. (2021). Noninvasive brain-machine interfaces for robotic devices. Ann. Rev. Control Robot. Auton. Syst. 4, 191–214. 10.1146/annurev-control-012720-093904

[B33] Vidaurre C. Blankertz B. (2010). Towards a cure for bci illiteracy. Brain Topogr. 23, 194–198. 10.1007/s10548-009-0121-6 19946737 PMC2874052

[B34] Vourvopoulos A. Bermúdez i Badia S. (2016). Motor priming in virtual reality can augment motor-imagery training efficacy in restorative brain-computer interaction: a within-subject analysis. J. Neuroeng. Rehabil. 13, 1–14. 10.1186/s12984-016-0173-2 27503007 PMC4977849

[B35] Vourvopoulos A. Ferreira A. i Badia S. B. (2016). Neurow: an immersive vr environment for motor-imagery training with the use of brain-computer interfaces and vibrotactile feedback, in International Conference on Physiological Computing Systems, volume 2 (Lisbon: SciTePress), 43–53. Available online at: https://www.scitepress.org/Link.aspx?doi=10.5220/0005939400430053

[B36] Vourvopoulos A. Jorge C. Abreu R. Figueiredo P. Fernandes J.-C. Bermudez i Badia S. (2019). Efficacy and brain imaging correlates of an immersive motor imagery bci-driven vr system for upper limb motor rehabilitation: a clinical case report. Front. Hum. Neurosci. 13, 244. 10.3389/fnhum.2019.00244 31354460 PMC6637378

[B37] WHO (2013). Fact-Sheet. World Health Organization. (Geneva). Available online at: https://www.who.int/news-room/fact-sheets/detail/cardiovascular-diseases-(cvds)

[B38] Wolpaw J. Wolpaw E. W. (2012). Brain-computer interfaces: principles and practice, in Oxford Scholarship Online (Oxford University Press). Available online at: https://academic.oup.com/book/1700

[B39] Zhang J. Wang M. (2021). A survey on robots controlled by motor imagery brain-computer interfaces. Cogn. Robot. 1, 12–24. 10.1016/j.cogr.2021.02.001

